# Triphenylphosphonium compounds preferentially inhibit long-chain fatty acid oxidation in cardiac mitochondria

**DOI:** 10.21203/rs.3.rs-9944913/v1

**Published:** 2026-06-19

**Authors:** Anna Faakye, Satoshi Matsuzaki, Venkateswararao Eeda, Vibhudutta Awasthi, Kenneth Humphries

**Affiliations:** Oklahoma Medical Research Foundation; Oklahoma Medical Research Foundation; University of Oklahoma Health Sciences Center; University of Oklahoma Health Sciences Center; Oklahoma Medical Research Foundation

**Keywords:** Mitochondrial targeting, triphenylphosphonium (TPP), bioenergetics, mitochondrial respiration, fatty acid oxidation

## Abstract

Triphenylphosphonium (TPP) is a lipophilic molecule widely used in targeting compounds into the mitochondria. Despite its wide use, TPP has known mitochondrial toxicity, the characteristics of which are not completely defined. In this study, we sought to determine if the effects of TPP and TPP conjugates on mitochondrial function occur in a substrate dependent manner. To do so, we treated isolated mouse heart mitochondria with TPP, commercially available TPP derivatives MitoTEMPO and MitoSOX, and a test compound (TPP-aspirin). All TPP conjugates, except MitoTEMPO which was relatively inert, preferentially inhibited mitochondrial respiration when it was supported by palmitoyl carnitine as compared to pyruvate. This substrate selectivity was not explained by differential effects on membrane potential or electron transport chain activities, as both were largely preserved at concentrations of compounds that inhibited respiration. To identify the site of inhibition, we measured fatty acid oxidation directly and found that TPP and its conjugates significantly inhibited β-oxidation activity in energized mitochondria. CPT1 activity was unaffected, localizing the inhibition to the inner mitochondrial compartment. Finally, acute treatment of AC16 cells with TPP showed the same preferential inhibition of oxygen consumption rates when comparing fatty acids to pyruvate, without the loss of cell viability. Cumulatively, these results show that TPP and TPP-conjugate effects on mitochondrial function have substrate dependency by targeting fatty acid oxidation.

## Introduction

Mitochondria are essential organelles responsible for a multitude of cellular processes, most notably the production of adenosine triphosphate (ATP) via oxidative phosphorylation. Beyond their role in bioenergetics, mitochondria are hubs for metabolic processes, central regulators of apoptosis, calcium homeostasis, and the generation of reactive oxygen species (ROS)[1, 2].

Mitochondrial dysfunction is a hallmark of numerous pathological conditions, including neurodegenerative disorders, cardiovascular diseases, cancer, and metabolic syndromes[3]. Consequently, the ability to specifically target mitochondria has significant implications for both basic research and therapeutic intervention. However, despite their importance, effective delivery of bioactive molecules to mitochondria remains a significant pharmacological challenge. The mitochondrial double membrane creates a selective barrier that limits the entry of many compounds[4, 5]. To overcome this, various mitochondrial targeting strategies have been developed, including mitochondria penetrating peptides, liposomes, and lipophilic cations[5–7].

Triphenylphosphonium (TPP) is one of the most widely used lipophilic cations for targeting drugs and probes to mitochondria[8, 9]. Its delocalized positive charge and hydrophobic phenyl rings structure enables efficient membrane permeation and selective accumulation in mitochondria without needing transporters[10, 11], making it a valuable tool for both research and therapeutic applications. TPP has been extensively used to deliver a range of bioactive cargos, including antioxidants (e.g. MitoTEMPO), redox sensors (e.g. MitoSOX), metabolic inhibitors, and other fluorophores[10]. The conjugation of drugs or probes to TPP enables selective mitochondrial localization, enhancing their efficacy and specificity. However, evidence also suggests that TPP is not merely an inert mitochondrial carrier. Several studies have reported that TPP itself, particularly at higher concentrations or with longer alkyl chains, can perturb mitochondrial function[8, 12]. Reported effects include dissipation of membrane potential, inhibition of respiratory chain complexes, alteration of mitochondrial morphology, and increased ROS production[8]. This is of particular importance in high demand tissues such as the heart, which relies on fatty acid oxidation for most of its ATP production but has extensive metabolic flexibility.

While TPP is widely utilized for mitochondrial targeting, its intrinsic bioactivity remains insufficiently characterized. TPP toxicity is known to be related to mitochondrial depolarization. Current literature often attributes TPP mediated dysfunction to non-specific membrane depolarization or detergent-like effects on the lipid bilayer. However, these models fail to explain potential variances in toxicity across different metabolic states. If TPP toxicity were purely a result of membrane disruption, one would expect a uniform decline in mitochondrial performance regardless of the fuel source. The possibility that TPP selectively interacts with specific metabolic pathways remains unexplored. In this study, we sought to investigate the direct effects of TPP and its derivatives on mitochondrial function. We demonstrate that fatty acid supported respiration, as compared to pyruvate supported respiration, is significantly more sensitive to the inhibitory effects of TPP. By delineating the concentration and substrate dependent effects of TPP on mitochondrial metabolism, our findings provide a critical framework for interpreting studies that utilize TPP conjugated probes, such as MitoSOX and MitoTEMPO, and guide its future application in mitochondrial drug delivery and toxicology research.

## Results

### TPP inhibits fatty acid-supported respiration.

**Figure 1:**
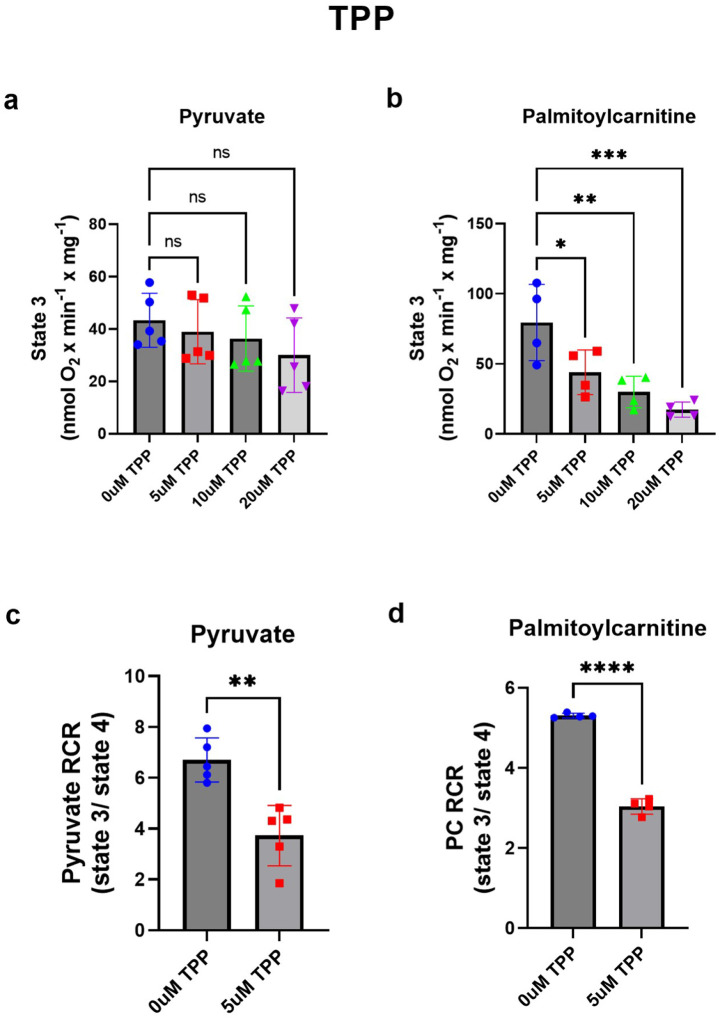
TPP inhibits fatty-acid supported mitochondrial respiration: **A**, Pyruvate-supported and **B**, PC-supported state 3 respiration rate of individually isolated mitochondria from mice treated with increasing concentrations of TPP (0–20μM) (*n* = 4–5 per group). **C-D**Respiratory control ratio (RCR, calculated as State3/State 4) of mitochondria from either **C** pyruvate/malate or **D** PC/malate in the presence of ADP (n = 4–5 per group). Data are presented as mean ± SD and analyzed using one-way ANOVA followed by Dunnett’s post hoc test **(A-B)** and unpaired Student’s *t* test **(C-D).** ns indicates not significant, *p < 0.05, **p < 0.01, ***p < 0.001****p < 0.0001

We first sought to establish the toxicity of TPP in an isolated mitochondrial system. Murine cardiac mitochondria were isolated and treated with increasing concentrations of TPP (5, 10 and 20μM). Isolated mitochondria were provided with either pyruvate or palmitoyl carnitine (PC), as these represent primary oxidizable nutrients of the heart. As shown in Fig. 1A, isolated mitochondria treated with increasing concentrations of TPP did not show a significant effect on pyruvate-supported mitochondrial state 3 respiration (the ADP-stimulated maximal respiration rate). However, TPP-treated mitochondria showed a significant dose-dependent decrease with PC-supported state 3 respiration (Fig. 1B), indicating a selective vulnerability of fatty acid metabolism.

To evaluate the integrity of the mitochondria and the efficiency of mitochondrial oxidative phosphorylation, the respiratory control ratio (RCR) was calculated. The RCR indicates how well mitochondria couple substrate oxidation to ATP synthesis[13, 14]. A lower ratio indicates less coupling efficiency and a higher RCR indicates that mitochondria are well coupled [13, 15]. Figure 1C&D shows TPP treated mitochondria had significantly lower RCRs whether pyruvate or PC were the respiratory substrate. Thus, TPP reduced coupling efficiency regardless of substrate. While this suggests that TPP reduces overall coupling efficiency, the substrate-specific decline in State 3 respiration points to a targeted inhibition of the fatty acid oxidation machinery rather than a purely non-specific membrane effect.

### TPP conjugates (MitoSOX, SATPP, and MitoTEMPO) impair pyruvate and fatty acid supported respiration.

**Figure 2:**
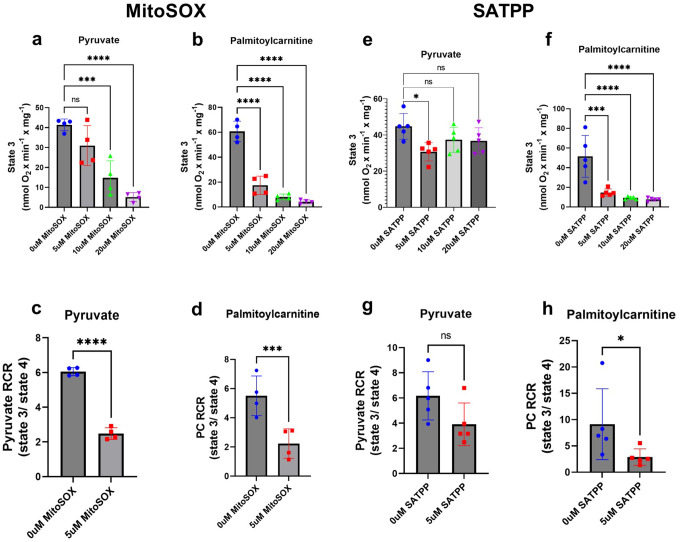
TPP derivatives, MitoSOX and SATPP inhibit fatty-acid supported mitochondrial respiration: **A, E**, Pyruvate-supported and **B, F**, PC-supported state 3 respiration rate of individually isolated mitochondria from mice treated with increasing concentrations of **A-B** MitoSOX or **E-F** SATPP (*n* = 4–5 per group). **C-D, G-H** Respiratory control ratio (RCR, calculated as State3/State 4) of mitochondria treated with MitoSOX or SATPP from either **C, G** pyruvate/malate or **D, H** PC/malate in the presence of ADP (n = 4–5 per group). Data are presented as mean ± SD and analyzed using one-way ANOVA followed by Dunnett’s post hoc test **(A, B, E, F)** and unpaired Student’s *t*test **(C, D, G, H).** ns indicates not significant, *p < 0.05, **p < 0.01, ***p < 0.001****p < 0.0001

Having established that TPP preferentially inhibits fatty acid supported mitochondrial respiration, we next examined if the substrate-specific inhibition was a general feature of TPP-targeted molecules. To do so we tested a panel of conjugates with chemically diverse cargos. We examined MitoSOX, a fluorescent probe used to detect mitochondrial superoxide; MitoTEMPO, a mitochondrial targeted antioxidant; and aspirin conjugated to TPP (SATPP), a test compound with cargo chemically distinct from MitoSOX and MitoTEMPO. The concentrations of the TPP-conjugated compounds used were between 5–20μM, as this is the concentration range where free TPP had inhibitory effects and also represent the typical experimental concentration ranges for MitoSOX and MitoTEMPO [16, 17]. Figure 2A&B shows the substrate specific effect of MitoSOX on mitochondrial respiration using pyruvate and PC respectively. MitoSOX caused a significant decrease in pyruvate supported state 3 (maximal) respiration at relatively higher concentrations (10 and 20μM). However, this inhibitory effect was more pronounced with PC as the substrate. 5uM, a standard working concentration of MitoSOX, was sufficient to significantly inhibit PC-supported, but not pyruvate-supported, state 3 respiration. However, in Fig. 2C&D the RCR was significantly decreased with all treatment groups (5–20μM) relative to control, irrespective of the substrate used. This suggests that metabolic artifacts may be present in studies using MitoSOX, even when general mitochondrial health, such as pyruvate respiration, appears intact.

Other conjugates of TPP were tested for their effects on mitochondrial respiration. As shown in Fig. 2.E&F, SATPP had similar mitochondrial effects as TPP and MitoSOX. State 3 respiration showed significant inhibition with PC with all the concentrations of SATPP treated as compared to the pyruvate-supported respiration, which was relatively less affected. Interestingly, the RCRs decreased with both pyruvate and PC- supported respiration regardless of the concentration of SATPP. (Fig. 2.G&H) likely a reflection of the known uncoupling activity of salicylic acid[18, 19]. We also investigated the effect of MitoTEMPO, a TPP conjugated antioxidant on mitochondrial respiration. Unlike free TPP and the other TPP conjugates, MitoTEMPO had no significant inhibitory effects at 5 or 10μM concentrations **(Supplementary Fig. S1 A&B)**. There was only a significant decrease in the PC-supported respiration at 20μM **(Supplementary Fig. S1 B)**. Likewise, the RCRs for isolated mitochondria treated MitoTEMPO were unaffected except with the highest dosage of MitoTEMPO and PC as the substrate **(Supplementary Fig. S1 C&D)**. Thus, despite the disparate nature of these cargos, overall, a consistent pattern of preferential FAO inhibition emerged, implicating the TPP targeting moiety in the observed toxicity.

### Acute TPP treatment does not affect mitochondrial membrane potential

**Figure 3:**
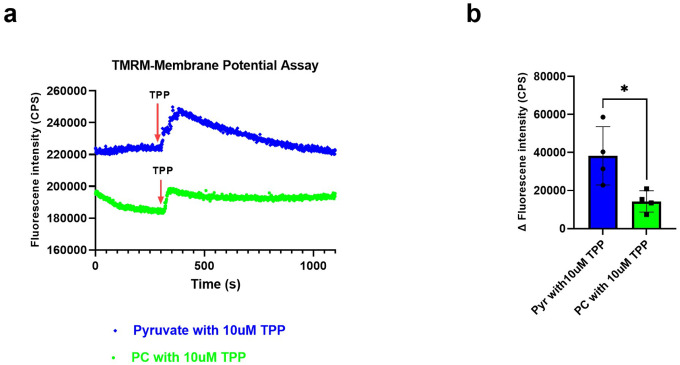
TPP effect on mitochondrial membrane potential. **A** is a TMRM response fluorescence intensity graph from the fluorospectrometric experiment. The blue and green traces represent isolated mitochondria with pyruvate or PC respectively as substrate. The red arrows show the time point at which 10μM TPP was added. Graph shown is an average of 4 fluorescence intensity recordings from 4 individually isolated mitochondria (n=4). **B** is the change in fluorescence between the peak intensity and the baseline (before TPP was added) n=4 per group. Data are presented as mean ± SD and analyzed using unpaired Student’s *t* test **(B)**. *p < 0.05

In addition to the lipophilic nature, the positive charge on the TPP moiety enables its accumulation to the mitochondria and this has been reported to contribute to the dissipation of the mitochondrial membrane potential[10]. We were therefore interested in determining if the substrate-dependent effect of TPP on mitochondrial respiration was mediated by differential effects on membrane potential. To do so, we used the membrane-permeable fluorescent dye TMRM which accumulates in mitochondria proportionately to the membrane potential. Experiments were performed in quench mode, whereby fluorescence decreases as the dye is accumulated in the mitochondria, but then fluorescence increases upon depolarization[20]. In Fig. 3A, which depicts the average of four biological replicates, isolated heart mitochondria were incubated with either pyruvate or PC substrates and TPP was added at 5 mins. The TPP induced a small and transient spike in fluorescence intensity (indicated by the red arrows in Fig. 3A) as some TMRM was released from mitochondria. Interestingly, the increase in fluorescence was greater with pyruvate than with PC as the substrate (Fig. 3B). The overall depolarization induced by 10 μM TPP was relatively modest, regardless of substrate used. This is shown by the addition of DNP, a classic mitochondrial uncoupler, which caused TMRM fluorescence to dramatically increase (indicated by the purple arrow) **Supplementary Fig. S2A**. The fact that 10 μM TPP induced a larger depolarization signal with pyruvate than with PC, the substrate most sensitive to respiratory inhibition, suggests that preferential dissipation of membrane potential is not the driver of TPP’s substrate-specific toxicity.

### TPP and TPP-conjugates do not alter NADH oxidase and Complex 1 activity

**Figure 4:**
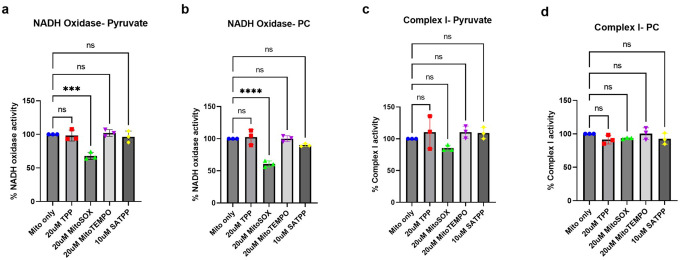
Effect of TPP on NADH oxidase and Complex I activity: **A-B** NADH oxidase activity of individually isolated mitochondria treated with TPP, MitoSOX, MitoTEMPO and SATPP in the presence of **A** pyruvate or **B**PC. **C-D** Complex I activity of individually isolated mitochondria treated with TPP, MitoSOX, MitoTEMPO and SATPP in the presence of **C** pyruvate or **D**PC. All results are expressed as the percentage of the activity of the untreated control and are presented as means ± SD, n = 3 per group. ns indicates not significant, ***p < 0.001, ****p < 0.0001

To determine if the substrate-specific inhibition was due to a direct blockade of the electron transport chain (ETC), we measured NADH oxidase activity, which provides a readout of the integrated function of Complexes I, III, and IV. Freshly isolated intact mitochondria were treated with either pyruvate or PC as substrates and TPP or its conjugates. Mitochondria were then disrupted by freeze thawing to perform the assays. As shown in Fig. 4A&B TPP, SATPP and MitoTEMPO showed no effect on NADH oxidase activity with either pyruvate or PC. Only MitoSOX treated mitochondria showed a significant decrease in NADH oxidase activity regardless of the substrate. The significant decrease in activity observed with MitoSOX suggests that this specific conjugate may have additional off-target effects on the ETC proteins, distinct from the primary TPP-mediated effect on fatty acid oxidation. We also measured Complex I activity, as it is the rate limiting step of the ETC. Its activity also showed a similar trend as NADH oxidase Fig. 4C&D, which showed no significant changes relative to the untreated control. These results demonstrate that the ETC remains functionally intact, providing evidence that TPP-mediated respiratory inhibition occurs upstream of the respiratory chain, likely at the level of substrate transport or initial oxidation.

### TPP and its conjugates inhibit fatty acid oxidation downstream of CPT1

**Figure 5:**
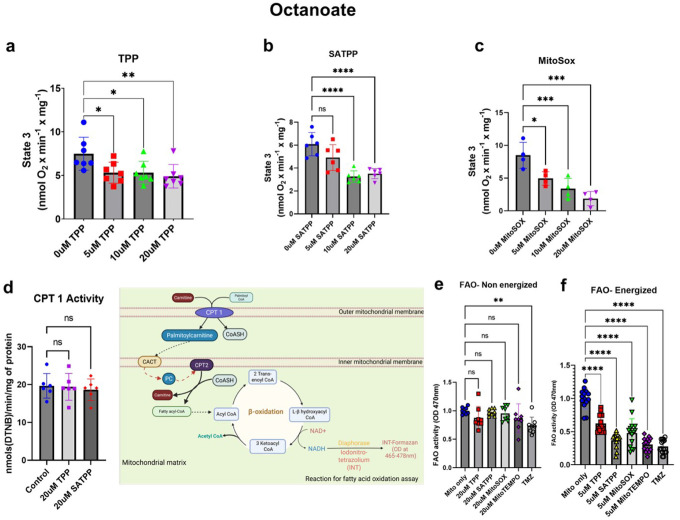
TPP effect on MCFA-supported respiration and fatty acid oxidation: **A-C**, Octanoate-supported state 3 respiration rate of individually isolated mitochondria from mice treated with increasing concentrations of TPP (A), SATPP (B) and MitoSOX (C) (*n*= 6/7 per group). **D**. CPT1 activity was measured from isolated mitochondria treated with 20 μM of TPP or SATPP (n=6 individually isolated mitochondria) **Scheme**, Graphical representation of the FAO reaction showing how NADH produced from β-oxidation reacts with diaphorase and INT to produce a red-colored INT-formazan measured at a spectral absorbance wavelength of 465–478nm. **E-F.** FAO activity was spectrophotometrically measured in isolated mitochondria treated with TPP, TPP-conjugates or TMZ (FAO drug inhibitor) **E** Activity was measured in mitochondria treated with TPP in a non-energized state (without PC) or **F** in an energized state (treated with TPP/TPP conjugates in the presence of PC). Each point represents an individual well from a 96-well plate, with the experiment performed on 2 or 3 separate plates from 3 individual biological replicates. Data are presented as mean ± SD and analyzed using one-way ANOVA followed by Dunnett’s post hoc test **(A-F)**ns indicates not significant, *p < 0.05, ***p < 0.001, ****p < 0.0001. (PC= Palmitoyl carnitine, CACT= Carnitine-acyl carnitine translocase, CPT 1/2= Carnitine palmitoyltransferase 1/2, INT= Iodonitrotetrazolium). (Scheme; Created in BioRender. Faakye, A. (2026) https://BioRender.com/bpeva9h)

The selective inhibition of PC-supported respiration, as compared to pyruvate-supported respiration, by TPP compounds could be due to inhibition of fatty acid transport into mitochondria. Long chain fatty acids require carnitine conjugates to be transported across the mitochondrial inner membrane. In our experiments with PC, we bypass CPT1 but rely on CPT2 to free the palmitate to support oxidative phosphorylation. In contrast to PC, shorter chain fatty acids can traverse the mitochondrial membrane as free acids independent of the CPT shuttling system. We next sought to investigate the effect of TPP and TPP-derivatives on mitochondrial respiration with the medium chain fatty acid (MCFA), octanoate. While the magnitude of inhibition compared to PC-supported respiration was less, Fig. 5A–C shows significant inhibition of state 3 respiration. Another difference between PC and octanoate was that RCRs showed no significant effects with TPP and MitoTEMPO but did so with SATPP and MitoSOX (**Supplementary Fig. S3 A-E**) at the higher concentrations. Thus, bypassing the CPT-shuttle system attenuates, but did not eliminate TPP-mediated respiratory inhibition. To more directly determine whether TPP is affecting the carnitine shuttle, we next measured CPT1 activity. As shown in Fig. 5D, treatment with either TPP or SATPP produced no significant changes in CPT1 activity, ruling out the outer membrane transport step as the site of inhibition.

We next measured fatty acid oxidation (FAO) activity, with palmitoyl CoA as the substrate, using a coupled assay (Fig. 5, **scheme**). For the assay, mitochondria were treated with 5μM or 20μM of TPP or TPP conjugates in the presence or absence of PC to energize the mitochondria. As validation of the assay, we also included a sample group treated with trimetazidine (TMZ). This is an inhibitor of long-chain 3-ketoacyl-CoA thiolase (3-KAT), the fourth step of the mitochondrial oxidation [21, 22]. After 5min of treatment, mitochondria samples were solubilized for the FAO assay. In the absence of an oxidizable substrate during the incubation, TPP and its conjugates had no significant effects on FAO (Fig. 5E). Only TMZ showed modest, but significant, inhibition. This reflects that without an oxidizable substrate, the resting membrane potential is insufficient for the uptake of TPP and TPP-conjugates and limits the uptake of TMZ. In contrast, when mitochondria were energized by coincubation with PC, 5μM TPP or TPP-conjugates caused a significant inhibition of FAO (Fig. 5F). The inhibitory effect of TMZ was also nearly complete under these energized conditions (Fig. 5F). Collectively, these results show that TPP and its conjugates directly inhibit FAO, but only when mitochondria are in an energized state.

### TPP impairs mitochondrial function in intact AC16 cardiomyocytes

**Figure 6:**
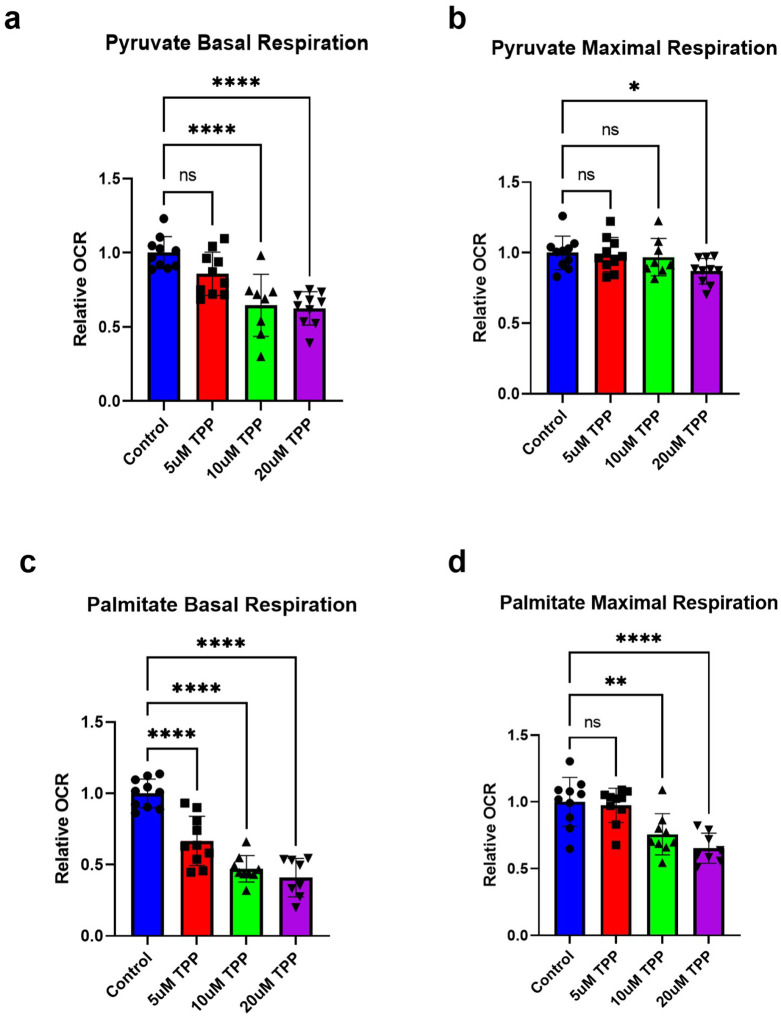
The acute response of cellular respiration to TPP in intact AC16 cells: **A&C.** Basal respiration rate (OCR, oxygen consumption rate) and **B&D.** Maximal respiration rate of AC16 cells treated with increasing concentration (5–20μM) of TPP for 10–15mins in the presence of pyruvate **(A&B)** or palmitate **(C&D)** as substrates. Each point represents an individual well from a Seahorse XFe24 plate, with the experiment performed on 2 separate plates. Data were normalized to cell numbers and are presented relative to untreated control. Data are shown as mean ±SD, ns indicates not significant, *p < 0.05, **p < 0.01, ***p < 0.001****p < 0.0001 by one-way ANOVA with multiple comparison of the mean of each test group to the mean of the untreated control.

We next sought to determine whether the substrate-dependent inhibition observed with isolated mitochondria translates to a more intact system. We evaluated the effects of TPP on mitochondrial respiration via Seahorse analysis using AC16 cells. This line is derived from the fusion of primary adult human ventricular heart with SV40 human fibroblasts [23, 24] and is widely used as a model system for investigating cardiac mitochondrial function[25–27]. As shown in Fig. 6A&B, when cells were provided with pyruvate, significant inhibition of basal and maximal respiration was only observed at the highest concentration of TPP (20μM). However, when cells were fueled by palmitate (Fig. 6C&D), TPP induced a significant, dose-dependent decrease in OCR, with inhibition observed at 5μM. This demonstrates that intact cells are significantly more sensitive to TPP when fueled by fatty acids compared to pyruvate[8]. This sensitivity to TPP was not due to the loss of cell viability. AC16 cells treated with an acute dose of 20μM TPP were co-stained with Hoechst 33342 and propidium iodide. We found no significant difference in viability between TPP and untreated cells (not shown), indicating that the observed respiratory effects represent a primary metabolic dysfunction rather than a loss of cell membrane integrity.

We also investigated the effects of longer-term TPP exposure. Following a 3-hour incubation, AC16 cells showed significant inhibition of both basal and maximal respiration regardless of the substrate or concentration used **(Supplementary Fig. S4**). This likely reflects the continuous accumulation of TPP over time, which eventually reaches a threshold that dissipates the mitochondrial membrane potential and causes generalized respiratory failure. Cumulatively, these data confirm that while TPP eventually causes broad mitochondrial dysfunction, the earliest and most sensitive manifestation of its toxicity is the specific impairment of fatty acid oxidation.

## Discussion

In this study we determined that TPP and several of its widely used conjugates inhibit the respiration of isolated cardiac mitochondria preferentially when PC, versus pyruvate, is the substrate. Using intact AC16 human cardiomyocytes, we validated that this substrate-specific vulnerability persists in an intact cellular environment, where TPP induced a decline in fatty-acid-supported oxygen consumption at concentrations that left pyruvate metabolism largely unaffected. Mechanistically, we show that this inhibition is not a secondary consequence of membrane potential dissipation or direct interference with the electron transport chain. Instead, the relative resilience of octanoate-supported respiration, combined with the inhibition of fatty acid oxidation, supports that TPP toxicity is mediated through inhibition of FAO.

Targeting drugs directly to the mitochondria has become a promising strategy for treating a range of diseases due to its central role in cell metabolism [28]. Mitochondria-targeted therapies exploit unique features of these organelles, such as their membrane potential, to selectively deliver drugs using carriers like triphenylphosphonium (TPP) cations, peptides, and nanoparticles, achieving concentrations up to 1000-fold higher within mitochondria compared to the cytosol [28–30]. While TPP is often viewed as an inert carrier, emerging evidence suggests it can exert detrimental effects on mitochondrial bioenergetics[10, 31]. Our findings extend this understanding by demonstrating that TPP-mediated toxicity is not a uniform suppression of function, but a substrate-specific impairment. To our knowledge, this is the first report to identify that the choice of metabolic fuel is a critical determinant of TPP’s inhibitory effects in cardiac mitochondria.

Previous research has shown that concentrations of TPP or its conjugates ranging from 1μM to 10μM have some level of inhibition of the mitochondrial electron chain. The degree of inhibition was increased by the hydrophobicity of the TPP derivative and the length of its alkyl chain linker [8, 10, 31]. Specifically, with isolated rat liver mitochondria, longer-chain alkyl TPP compounds had significant inhibitory effects on both Complex I and III activities whereas shorter chain TPP conjugates did not[8]. Additionally, TPP and other TPP derivatives have been shown to reduce the level/activity of Complex I holocomplexes and ETC supercomplex content with chronic (96h) treatment in vitro [32]. In the present study we did not observe inhibition of Complex I activity for TPP or any of its derivatives examined (Fig. 4C–D). This could be due to the relatively greater polarity of the conjugates we examined and the brief duration of treatment. Notably, we only observed inhibition of NADH-oxidase activity with MitoSOX, which occurred irrespective of the substrate (PC or pyruvate) used (Fig. 4A–B). MitoSOX has been previously reported to exhibit toxic effects that uncouple mitochondria and inhibit Complex I and IV at lower (5–10μM) concentrations [33]. While we did not observe such inhibition of Complex I, our results do support the capacity of MitoSOX to impair overall ETC activity. Unlike TPP and other derivatives explored in this study, MitoTEMPO did not show significant mitochondrial substrate inhibition. This is in accordance with previous literature that has shown MitoTEMPO exerts fewer toxic effects on mitochondrial function compared to other derivatives of TPP [34, 35]. MitoTEMPO functions to neutralize superoxide and reduce mitochondrial ROS without affecting cytosolic ROS. These properties help to preserve mitochondrial membrane potential and maintain ATP production [35]. Nevertheless, we do see that MitoTEMPO had similar inhibition of FAO as the other conjugates. We posit that once the mitochondrial membrane is disrupted to assay the enzymes, there is direct access to inner mitochondrial proteins that may account for the significant FAO inhibition by all conjugates (Fig. 5F).

Mitochondrial membrane potential represents the electrical component that drives ATP generation, and its measurement serves as a key indicator of mitochondrial health and function. Previous studies have shown that TPP and its derivatives, depending on their structure and hydrophobicity, can depolarize mitochondria [8, 36]. However, our current findings showed that isolated mitochondria treated acutely with TPP had a minimal effect on membrane potential (Fig. 3). Crucially, this stability was observed regardless of the respiratory substrate used, even under conditions where PC-supported respiration was significantly inhibited. This suggests with short term treatment, selective inhibition of fatty acid oxidation occurs through inhibition of the fatty acid oxidation machinery, while longer durations of treatment likely lead to TPP accumulation and membrane potential dissipation or ETC inhibition.

Several lines of experimentation support that acute TPP exposure inhibits mitochondrial respiration with selectivity towards fatty acid supported respiration. However, we did find that in AC16 cells, longer treatment with TPP had a dramatic inhibitory effect on oxygen consumption rate irrespective of provided oxidizable substrate (**Supplementary Fig. S4**). Under normal conditions, heart mitochondria primarily utilize long-chain fatty acids (LCFAs) such as palmitate for ATP production. LCFAs are transported into the mitochondria via the carnitine shuttle system (CPT1, CACT, CPT2), after which β-oxidation generates NADH and FADH_2_ for the ETC [37, 38].

The partial attenuation of inhibition with octanoate, a medium-chain fatty acid that bypasses the carnitine shuttle, supported that inhibition was primarily occurring downstream of the transport machinery. More directly, our FAO assay using palmitoyl CoA in solubilized mitochondria, which bypasses the carnitine shuttle entirely, confirmed that β-oxidation enzymes themselves are targets.

The data presented here provides caution when considering both experimental and therapeutic applications of TPP and its conjugates. For tissues with high fatty acid reliance, such as the heart, even low micromolar concentrations of TPP-conjugated probes may introduce confounding metabolic disruptions that are not captured by standard bioenergetic readouts such as pyruvate-supported respiration. The use of probes such as MitoSOX should consider including fatty acid-supported measurements as a control for off-target metabolic effects, particularly when working at concentrations at or above 5μM. More broadly, the relative sparing observed with MitoTEMPO shows that the chemical nature of the cargo is an important consideration. These observations suggest that next-generation mitochondrial carriers could be designed to minimize interactions with the fatty acid oxidation machinery. These findings should be considered in the evaluation of existing mitochondria-targeted compounds and in the development of new diagnostic probes or therapeutic agents.

## Experimental Procedures

### Animals

C57Bl6J mice were obtained from The Jackson Laboratory and bred in the Oklahoma Medical Research foundation (OMRF) animal facility. Mice were used in accordance with protocols approved by the OMRF Institutional Animal Care and Use Committee (IACUC). Experimental procedures were carried out in approved animal facilities at OMRF. Mice used for this study were 10–12 months old.

### Materials

All chemicals were purchased from Sigma-Aldrich unless stated otherwise. MitoSOX (M36007) was purchased from Thermo Fisher Scientific and dissolved in DMSO to make a stock solution of 5mM. TPP used for the assay was 4(Bromobutyl)triphenylphosphonium bromide (272132), TMZ was trimetazidine dihydrochloride (T7133) from LKT laboratories and MitoTEMPO was (2-(2,2,6,6-Tetramethylpiperidin-1-oxyl-4-ylamino)-2-oxoethyl)triphenylphosphonium chloride (SML0737); for the synthesis of SA-TPP chemicals, reagents were purchased from Millipore Sigma.

### Synthesis of SATPP: (5-(2-carboxyphenoxy)-5-oxopentyl)triphenylphosphonium.

SATPP was synthesized as shown in **Scheme 1** under these conditions: (i) Pyridine, at room temperature for 24h (8% yield); (ii) MgSO4, H2SO4, t-Butanol (80% yield); (iii) Triphenyl Phosphine, Toluene reflux for 12h (63% yield); 6N HCl, acetonitrile, reflux for 1h (80% yield after recrystallization in Ethanol). Characterization of SATPP by NMR was done by using JEOL JNM-ECZR 500MHz.

### Isolation of Cardiac Mitochondria

Mitochondria were isolated via differential centrifugation as previously described[39]. Briefly, 10–12 months old C57Bl6J mice were euthanized by cervical dislocation. Their hearts were perfused following the opening of the chest cavity with 5 ml of ice-cold isolation buffer containing 210 mM mannitol, 70 mM sucrose, 1.0 mM EDTA (dipotassium salt), and 5.0 mM 3-(N-morpholino)propanesulfonic acid (MOPS) adjusted to pH 7.4 with KOH. Hearts were excised and placed into 5 ml of Isolation buffer in a 15mL homogenizing tube. This was followed by five passes with a motor-driven Potter-Elvehjem tissue grinder. The homogenate was spun at 550 ×*g* for 5 min at 4°C, and the supernatant was collected, passed through a cheesecloth, and spun again at 10000 × *g* for 10 min. The resulting mitochondrial pellet was resuspended in ~60μl of isolation buffer, and the protein concentration was determined by the BCA (bicinchoninic acid) method (Thermo Fisher Scientific) using bovine serum albumin (BSA) as a standard according to [40].

### Mitochondrial respiration measurements

Following mitochondrial isolation and suspension, respirometry was performed as previously described[15]. Briefly, mitochondria were diluted to 0.25 mg/mL in OXPHOS buffer (210 mM mannitol, 70 mM sucrose, 10 mM MOPS, and 5 mM K_2_HPO_4_, pH 7.4), with 0.5 mg/mL BSA. For TPP and TPP-conjugates (MitoTEMPO, MitoSOX and SATPP) treatment experiments, mitochondria were diluted to 0.25mg/ml in OXPHOS buffer with 0.5mg/ml BSA with the indicated concentrations (5–20μM) of TPP or TPP-conjugates for 5mins at room temperature. The indicated oxidizable substrates, either 0.1-mM pyruvate and 1.0-mM malate, 25-μM palmitoyl carnitine (PC) and 1.0-mM malate or 0.1-mM octanoate and 1.0-mM malate were added to initiate state 2 respiration. Oxygen consumption was measured at room temperature using a fluorescence lifetime-based dissolved oxygen monitoring system (Instech). Following state 2 respiration, 0.5-mM ADP was added at 2 min to induce state 3 respiration. State 4 respiration was calculated as the slower, linear rate of oxygen consumption that occurred approximately 5 min after the initiation of state 3.

### Measurement of NADH oxidase/complex I activity

NADH oxidase activity, representing overall electron transport chain (ETC) function through complexes I–III–IV, was measured spectrophotometrically as previously described[41]. Briefly, isolated mitochondria were diluted to 0.25mg/mL in OXPHOS buffer with 0.5mg/mL BSA. The indicated oxidizable substrates, 0.1mM pyruvate and 1.0mM malate, or 25μM PC and 1.0mM malate, were added with 20uM of indicated TPP or TPP-conjugates (MitoTEMPO, MitoSOX and SATPP) for 5mins. The samples were immediately flash-frozen. Frozen-thawed samples were diluted to 0.01 mg/mL in 25 mM MOPS buffer with 10mM KCl (pH 7.4). The rotenone-sensitive rate of NADH oxidation was monitored at 340 nm using an Agilent 8453 UV–Vis spectrophotometer following the addition of 150μM NADH. Complex I activity was determined by measuring NADH oxidation in the presence of 50nM antimycin A and 100μM ubiquinone-1. Reactions were initiated by adding 150μM NADH, and the rate of oxidation was recorded at 340nm. This activity was dependent on the presence of ubiquinone-1 and was completely inhibited by 100nM rotenone, confirming its reliance on complex I function. Activities were calculated using the extinction coefficient of ε_340_ = 6200 M^−1^·cm^−1^ for NADH.

### CPT1 activity

CPT 1 activity was measured spectrophotometrically as previously described [41, 42]. Isolated mitochondria were diluted to 0.25mg/mL in OXPHOS buffer with 0.5mg/mL BSA. To this, 25μM PC and 1.0mM malate were added with 20 μM of TPP or SATPP and snap frozen. 100μL of this frozen-thawed mitochondria suspension, 100 μM palmitoyl CoA, and 100μM 5,5-dithio-bis-(2-nitrobenzoic acid) (DTNB) were preincubated for 20 minutes in a CPT assay buffer (25 mM MOPS at pH 7.4, 1 mM EGTA, 0.1% BSA). After acquiring a baseline measurement, CPT1 activity was measured spectrophotometrically for 10 minutes as the rate of increase at 412nm (wavelength) upon the addition of carnitine (10mM final). One sample assayed without carnitine was subsequently used to subtract the background, carnitine-independent, activity.

### Fatty-Acid Oxidation (FAO) Assay

FAO was measured using a colorimetric assay via iodonitrotetrazolium (INT) and diaphorase. Briefly, isolated mitochondria were diluted to 1 mg/mL in OXPHOS buffer with 0.5 mg/mL BSA. For energized FAO analysis, 25μM PC was added to isolated mitochondria and cotreated with 5μM TPP or TPP conjugates for 5mins at room temperature. However, for non-energized FAO analysis, isolated mitochondria were treated with 20μM TPP or TPP-conjugates without PC for 5mins at room temperature. As a positive control, isolated mitochondria were also treated with 100μM TMZ, a well described FAO inhibitor [21, 22] under the same conditions. This treated-mitochondria suspension was diluted to 0.25 mg/mL in FAO buffer (25mM MOPS at pH 7.4, 0.05% triton X-100) containing 100μM INT, 10unit/mL diaphorase, 250μM NAD^+^ and 50μM palmitoyl CoA (final concentrations) and incubated in a non-CO_2_ incubator for 1hr in a 96-well plate. Palmitoyl CoA added is oxidized to generate NADH from the fatty-acid oxidation cycle which reacts with the INT to produce a cherry-red colored formazan that was measured spectrophotometrically at an absorbance spectrum of 465–478nm. Control well reading (without the addition of palmitoyl CoA) was subtracted from reaction well reading for each sample. The subtracted optical density (O.D.) reading is proportional to fatty acid oxidation activity of the sample.

### Mitochondrial Membrane Potential measurements

Membrane potential was measured using tetramethylrhodamine, methyl ester (TMRM, Thermo Fisher). Isolated mitochondria diluted to 0.25mg/mL in OXPHOS buffer with 0.5 mg/mL BSA and the indicated oxidizable substrates (0.1mM pyruvate and 1.0mM malate, or 25μM PC and 1.0mM malate) were incubated with 0.5mM TMRM and fluorescence monitored at 549/575 nm with a FluoroMax-4 spectrofluorometer. TPP was added after 5min of baseline reading and the fluorescence intensity was recorded. To determine the maximum membrane potential capacity, (2,4-dinitrophenol) DNP, an uncoupler was added after 10minutes and fluorescence intensity was recorded.

### Cell culture

AC16 cells which are proliferating human cardiomyocytes cell line derived from the fusion of primary adult human ventricular heart with SV40 human fibroblast[23, 24] were purchased from Millipore (SCC109). These cells display protein expressions and metabolic profiles comparable to those of primary human cardiomyocytes [23, 24]. AC16 cells were cultured in Dulbecco’s Modified Eagle’s Medium/Nutrient Mixture F-12 Ham (DMEM/F12- Sigma) containing 3.15g/L glucose and supplemented with 2mM L-glutamine (Sigma), 12.5% Fetal Bovine Serum (FBS- Sigma) and 1X penicillin-streptomycin solution (Sigma). Cells were subcultured in 10cm dish at 37°C and 5% CO_2_ in an incubator with media change done every 2–3 days. Cells were passaged when they reached 80–90% confluency. Cells from identical passages were used for each experiment (cells from passages 7–13 were used for all experiments).

### Seahorse XF analysis

Mitochondrial oxygen consumption was measured using the Agilent Seahorse XF24 Extracellular Flux Analyzer. (Seahorse Bioscience). AC16 cells were plated overnight in a 24-well Seahorse XF24 plate after 40 minutes of laminin coating at a density of 15000 cells per well. At 90–95% confluence, media was changed to un-buffered DMEM and the plate incubated in a non-CO_2_ incubator (37°C; 60 min) before running on the XF24 Analyzer [43]. Palmitate-BSA conjugate cocktail was made by incubating 90%BSA:10% 50mM palmitate for 1h in a non-CO_2_ incubator. Then 1mM Pyruvate/BSA or 0.1mM palmitate/BSA substrates were added to the cells after the 1hr starvation, with or without TPP, right before plate was placed in the Seahorse analyzer for acute treatment conditions. For chronic treatment, cells were treated with TPP 3hours prior to Seahorse measurements. Oxygen consumption rate (OCR) was read over the course of 3 measurement cycles. Each measurement cycle consists of 50sec of mixing, 1:30 min of wait and 2:30 min of measurements. Oligomycin A (OA, 1μM), carbonyl cyanide-p-trifluoromethoxyphenylhydrazone (FCCP, 4μM), and antimycin A (AA, 1μM) were added in three sequential injections. All experiments were conducted twice, and viability was assessed by Hoechst 33342 staining. Additionally, cells were co-stained with propidium iodide to assess cell viability. OCR data was normalized to cell number using an Agilent BioTek Cytation 5 cell imager.

### Statistical Analysis

Data was analyzed using GraphPad Prism (Version 9). One-way ANOVA was used to compare control and treated groups with Dunnett’s multiple comparisons test for post-hoc analysis. All results are expressed as means ± SD unless otherwise indicated with *n* representing the number of individual experiments. Statistical significance was established at *P* ≤ 0.05 and indicated in each figure legend. (*p < 0.05, **p < 0.01, ***p < 0.001, ****p < 0.0001)

## Supplementary Material

This article contains supporting information

Supplementary Files

This is a list of supplementary files associated with this preprint. Click to download.

- SupplemenataryInformation2.docx

## Data Availability

All data are contained within the manuscript

## Data Availability

All data are contained within the manuscript.

## Abbreviations

Non-Standard Abbreviations.

TPP: Triphenylphosphonium
MOPS: 3-(N-morpholino)propanesulfonic acid
DTNB 5: 5-dithio-bis-(2-nitrobenzoic acid)
PC: Palmitoyl carnitine
TMRM: Tetramethylrhodamine methyl ester
RCR: Respiratory control ratio
DNP: 2,4-dinitrophenol
OCR: Oxygen consumption rate
FCCP: Carbonyl cyanide-p-trifluoromethoxyphenylhydrazone
ETC: Electron transport chain
CPT1/2: Carnitine palmitoyl transferase 1/2
MCFA: Medium chain fatty acid
CACT: Carnitine-acyl carnitine translocase
LCFA: Long chain fatty acid
FAO: Fatty acid oxidation
ATP: adenosine triphosphate
NADH: Nicotinamide adenine dinucleotide (reduced)
FADH: Flavin adenine dinucleotide
NMR: Nuclear magnetic resonance
TMZ: Trimetazidine
INT: Iodonitrotetrazolium
